# Early steps in inner ear development: induction and morphogenesis of the otic placode

**DOI:** 10.3389/fphar.2015.00019

**Published:** 2015-02-10

**Authors:** Xiaorei Sai, Raj K. Ladher

**Affiliations:** Laboratory for Sensory Development, RIKEN Center for Developmental BiologyKobe, Japan

**Keywords:** inner ear, induction, FGF signaling, myosin-II, morphogenesis

## Abstract

Various cellular replacement therapies using *in vitro* generated cells to replace damaged tissue have been proposed as strategies to alleviate hearing loss. All such therapies must involve a complete understanding of the earliest steps in inner ear development; its induction as a thickened plate of cells in the non-neural, surface ectoderm of the embryo, to its internalization as an otocyst embedded in the head mesenchyme of the embryo. Such knowledge informs researchers addressing the feasibility of the proposed strategy and present alternatives if needed. In this review we describe the mechanisms of inner ear induction, concentrating on the factors that steer the fate of ectoderm into precursors of the inner ear. Induction then leads to inner ear morphogenesis and we describe the cellular changes that occur as the inner ear is converted from a superficial placode to an internalized otocyst, and how they are coordinated with a particular emphasis on how the signaling environment surrounding the inner ear influences these processes.

## INTRODUCTION

The inner ear is a complex structure. It is composed of numerous cell types that include ciliated mechanoreceptors to detect mechanical stimuli associated with balance or sound detection, the neurons of the eight cranial nerve, the cochleovestibular nerve, that transmits the stimuli to the central nervous system, as well as other cell types to maintain the unique ionic composition of the inner ear (Slepecky, 1996; Highstein et al., 2004). All of these cell types are derived from the otic placode, a seemingly simple epithelial structure that is part of the surface non-neural ectoderm adjacent to the caudal part of the hindbrain (Barald and Kelley, 2004; Groves and Fekete, 2012). As is typical for most tissue induction during early development, secreted factors from surrounding tissue act on competent ectoderm to convert its fate (Ladher et al., 2010). This dynamic epigenetic control ensures that the otic placode forms at the right time and the right place, starting the train of events that will convert the placode into a fully functioning inner ear. The otic placode is induced superficial, yet the inner ear itself is embedded within the cephalic mesenchyme (Meier, 1978a,b). Thus, morphogenetic processes must take place that internalize this tissue. The process, called invagination occurs concomitantly with induction. Thus, changes in the shape of the cells are intimately linked to the inductive process. Indeed, changes in the cytoskeletal architecture of the otic placode are directly controlled by secreted factors, and it is clear that for complete and accurate morphogenesis the topographical relationship of the otic placode to the tissue emitting the inducing signal is critically important (Sai and Ladher, 2008).

Culture studies of isolated otic placodes at different stages have shown that once induction and invagination is complete, sensory cell and neuronal differentiation is autonomous in this tissue (Freter et al., 2008). This suggests that these early events by themselves are sufficient for at least some later differentiation. Thus, a deep understanding of the earliest events in inner ear development is necessary to inform cell differentiation protocols and attempts *in vitro* organ engineering that will form the next-generation of therapies for hearing loss.

## INDUCTION

The otic placode is part of a series of cranial placodes located in the head of all vertebrate animals. Placodes are thickened regions of ectoderm adjacent to the neural plate boundary that will give rise to sensory structures in the head as well as many of the cranial nerves (Baker and Bronner-Fraser, 2001; Schlosser, 2006, 2014; Graham and Shimeld, 2013). Anterior is the olfactory placode, which will generate the nasal epithelium as well as the first cranial nerve. Caudal to the olfactory placode is the lens placode. Uniquely, amongst the cranial placode, it produces neither neurons nor sensory cells. The two lobes of the trigeminal placode, the ophthalmic and the maxilla-mandibular are found around the eye. These placode are wholly neurogenic and form the sensory neurons of the fifth cranial nerve. As already stated, the otic placode will give rise to the inner ear as well as the eight cranial nerve. Lateral to the otic placode are the epibranchial series of placodes, the most anterior, the geniculate, forms the sensory components of the seventh cranial nerve, innervating the taste buds and conveying touch information from the ear lobes. The second epibranchial placode (EPD), the petrosal, will give rise to the ninth cranial nerve, innervating the tongue, as well as the carotid body. The most caudal is the nodose EPD, and will contribute to the tenth cranial nerve, the vagus (Baker and Bronner-Fraser, 2001; Ladher et al., 2010).

**FIGURE 1:**
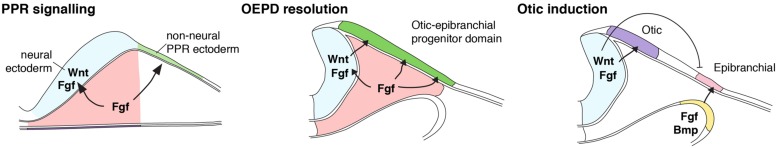
**Model of inner ear induction.** Shown is a scheme summarizing the induction of the inner ear, synthesizing data from zebrafish, chick, and mouse. At early neurulation stages, mesodermal source of FGF signals overlying pre-placodal ectoderm to adopt an otic-epibranchial placode (OEPD) fate. Mesodermal FGF also signals neural ectoderm to express FGF and Wnt signals. At later neurulation stages, soon after the neural tube has closed, Wnt then acts on OEPD to specify the inner ear from within the OEPD. (Modified from Ladher et al., 2010).

The otic placode, like all the cranial placodes, arises from the pre-placodal region (PPR); this can be thought of as ectoderm that is competent to develop into any placode given the correct signals, but has not yet committed to one (Bailey and Streit, 2006; Groves and LaBonne, 2014; Saint-Jeannet and Moody, 2014). Otic placode induction occurs by signals from adjacent tissues acting on the PPR. Otic induction itself can be though of as a progressive process, with a gradual restriction of possible fates as a function of time, under the control of signaling interactions (**Figure 1**). This section describes these processes, and the signals and transcription factors that control them, in more detail.

### ESTABLISHING THE PRE-PLACODAL REGION

One of the first events in the patterning of the embryonic ectoderm is its separation into neural and non-neural domains in the head region of the embryo. This occurs at around the time of gastrulation and is directed by signals coming from the mesoderm and endoderm as well as signals acting within the ectoderm itself (Ahrens and Schlosser, 2005; Litsiou et al., 2005; Bailey and Streit, 2006). Subsequent interactions cause the border region between these two territories to become a region of competence for two different types of tissue; the region of the border within the neural plate is, given the right set of signals, able to generate neural crest. In contrast, border cells that are found in the non-neural ectoderm are competent to generate sensory placodes, and this is called the pre-PPR (Pieper et al., 2012).

Molecularly the PPR is defined by the expression of Six and Eya family transcription factors (Schlosser, 2007; Sato et al., 2012; Groves and LaBonne, 2014; Saint-Jeannet and Moody, 2014; Yajima et al., 2014). These describe a strip of expression wrapped around the neural plate, from roughly the first somite to the rostral tip of the neural plate. Mutations in these genes do lead to deficits in some placodal derivatives, but do not result in a complete absence of the placodes (Oliver et al., 1995; Ozaki et al., 2002, 2004; Laclef et al., 2003; Zheng et al., 2003; Konishi et al., 2006; Zou et al., 2006; Ikeda et al., 2007, 2010; Li et al., 2010; Suzuki et al., 2010a,b; Yajima et al., 2010). This could be due to redundancy amongst the Eya and Six family transcription factors, or it could suggest that the exact nature of the PPR is a more complicated than simply a region of competence for all placodes. The importance of the PPR in otic induction was demonstrated in the experiments of Martin and Groves (2006). In these experiments, competence of ectoderm to express the otic induction marker *Pax2* in response to FGF2 was tested. The finding that ventral ectoderm could only respond if transplanted to the PPR/neural border region for 6 h strongly supports the idea that the PPR should be considered a region of competence to respond to placode inducing signals. Furthermore this competence is actively conferred.

Embryological experiments have suggested that the PPR is induced from signals that come from surrounding tissues (Ahrens and Schlosser, 2005; Litsiou et al., 2005; Bailey and Streit, 2006). In chick, the ablation of the head mesoderm abolishes the expression of PPR markers (Litsiou et al., 2005). Conversely, head, but not trunk mesoderm induces their expression in non-neural ectoderm. *Fgf4* and *Fgf8* are both expressed in the mesoderm that underlies the PPR, making them likely candidates to act as PPR inducing signals (Ahrens and Schlosser, 2005; Litsiou et al., 2005). Indeed, inhibition of FGF signaling can down-regulate some markers of the PPR in chick (Litsiou et al., 2005) and in embryos of the amphibian *Xenopus* (Ahrens and Schlosser, 2005). Furthermore, FGF8 protein is able to induce some PPR markers in competent non-neural ectoderm (Litsiou et al., 2005), however, it remains to be seen if this tissue is then competent to respond to FGF2 to induce otic markers. The cranial mesoderm also expresses inhibitors of Wnt and BMP (bone morphogenetic protein) signaling, suggesting that the activity of these proteins is inhibitory to PPR formation. Indeed, activation of Wnt or BMP signaling does inhibit the expression of PPR markers, whereas the inhibition of these signals can expand PPR marker gene expression. Thus it is likely that FGF signaling in combination with Wnt and BMP inhibition is necessary for complete PPR induction (Litsiou et al., 2005). What is not clear is the hierarchical organization of these signals; it is possible that some of these signals act upstream to promote the differentiation of non-neural ectoderm and that other signals confer PPR fate secondarily. One possible way of resolving this is to use *in vitro* cell systems, such as ES cells, to monitor the exact tissue induced by each kind of treatment over fine time points. If combined with a PPR specific reporter line (Sato et al., 2010, 2012), this experiment would also serve to greatly optimize the induction of inner ear tissues in ES cells.

As we will describe in the next section, FGF signaling is reused in the formation of the inner ear precursor domain. It is clear that FGF signaling has different consequences over a very narrow time window, and this raises the conundrum of how these responses are coordinated and spirited in time. One possible mechanism is by invoking the idea of feed-forward loops. Here, one can suggest that the action of FGF signaling in PPR induction also induces a transcription factor that is able to alter the response to FGF signaling. One example may be the *Foxi3* transcription factor. *Foxi3* is necessary for the response of PPR ectoderm to FGF signals to express *Pax2* (Khatri et al., 2014). Interestingly, the expression of *Foxi3* is controlled by the same tissues that control the expression of other PPR markers, suggesting not only a mechanism that ensures the correct temporal response to FGF signaling but also adds a molecular correlate to the idea that the PPR is a region of ectoderm competent to respond to placode inducing cues.

### INDUCTION OF THE OTIC-EPIBRANCHIAL PROGENITOR DOMAIN

The mechanisms underlying the formation of the inner ear have been actively investigated for over 100 years. In this time researchers have used a variety of model systems, namely amphibians, fish, birds and mammals, and a variety of approaches, from experimental embryology to genetic studies, to ask how otic induction is controlled (reviewed in Ladher et al., 2010; Groves and Fekete, 2012). This work has provided a consensus view on how the posterior part of the PPR is specified to the inner ear fate.

One of the earliest markers that are induced during otic induction is the transcription factor *Pax2* or, depending on the species, its close homolog *Pax8* (Christophorou et al., 2010; Freter et al., 2012). *Pax2/8* expression commences at mid-neurula stages (when the neural tube is closing) next to the caudal part of the future hindbrain in all vertebrate species that have been investigated (Plachov et al., 1990; Stoykova and Gruss, 1994; Lun and Brand, 1998; Pfeffer et al., 1998; Funahashi et al., 1999; Kozmik et al., 1999; Bouchard et al., 2002; Krelova et al., 2002; Hans et al., 2004; Mackereth et al., 2005). This location is at the junction of the head and the trunk of the animal, and extends anterior to the first somite. Lineage labeling of the *Pax2* domain revealed that expression encompasses progenitors of not only the inner ear but also the EDPs (Bouchard et al., 2004; Ohyama and Groves, 2004; Streit, 2004; McCarroll et al., 2012). Thus, it is clear that the initial step of PPR restriction is the induction of a common progenitor domain for the otic and EDPs. This domain has been termed the otic-epibranchial progenitor domain (OEPD), the pre-otic field (POF), or the posterior placodal area (PPA). In this review, we will use the OEPD terminology.

Embryological experiments, defined the region of the embryo that was able to induce the OEPD. In particular, embryos from chick and the amphibian *Xenopus* showed that paraxial mesoderm plays a key role (reviewed in Ladher et al., 2010; Groves and Fekete, 2012). Chick experiments were able to resolve this domain to the mesoderm between the first somite and the level of the third rhombomere (Groves and Bronner-Fraser, 2000; Ladher et al., 2000; Kil et al., 2005). This underlies with the ectodermal domain of *Pax2/8*. However, it is also clear that the mesoderm is not the only source for signals. *Pax2* can only be induced in non-neural ectodermal tissue by mesoderm when some neural ectoderm is also included; without this neural contribution, the OEPD is not induced (Ladher et al., 2000). Thus, it is likely that both mesodermal and neural ectodermal signals contribute to the induction of the OEPD.

Several lines of evidence suggest that members of the fibroblast growth factor (FGF) family mediate the induction of the OEPD (reviewed in Schimmang, 2007). In chick, knockdown studies (using antisense oligonucleotide or neutralizing antibodies) pointed to a role for FGF3 in OEPD induction (Represa et al., 1991; Vendrell et al., 2000). Indeed the expression of *Fgf3* in the mesoderm and hindbrain of the chick embryo is consistent with it playing a major role in OEPD induction (Mahmood et al., 1995; Freter et al., 2008). However, knockouts in the mouse and further experiments in the chick seem to rule out an exclusive role for FGF3. In mouse *Fgf3* mutants the inner ear is induced although later development is impaired (Mansour et al., 1993). In the mouse, *Fgf3* is not expressed in the mesoderm and this has led to the idea that perhaps one or more FGF ligands are also required to mediate OEPD induction. In the chick, *Fgf19* is expressed in a similar pattern to *Fgf3*, appearing first in the mesoderm and then in the hindbrain (Ladher et al., 2000). Knockdown of just *Fgf19* has no real effect, however, when both *Fgf19* and Fgf3 are knocked-down, OEPD induction does not take place (Freter et al., 2008). In the mouse, *Fgf10* is expressed in the mesoderm, apparently taking the placode of chick Fgf19, and mutants of both *Fgf3* and *Fgf10* fail to induce the OEPD (Wright and Mansour, 2003).

Control of mesodermal *Fgf10* (in the mouse) or *Fgf19* (in the chick) seems to be, itself, under the control of signaling from the underlying endoderm, in this case by yet another member of the FGF family, *Fgf8*. The OEPD fails to be induced in mutants for *Fgf3* and *Fgf8*. The situation is slightly different in the chick, where *Fgf8* knockdown alone appears necessary for OEPD induction (Ladher et al., 2005).

One open question is what does the OEPD represent? Are OEPD cells equipotent, with individual cells able to give rise to both otic and epibranchial progenitors? Is it a mixed population of interspersed epibranchial and otic progenitors? Or are otic and epibranchial progenitors confined to distinct domains within the OEPD? Individual cell labeling and tracing will resolve this question, however, some clues exist when expression patterns are inspected. In particular the expression of Foxi1 is noteworthy. In chick *Foxi1* is expressed around the OEPD at the time of OEPD induction, however, it’s expression begins to encroach into the OEPD such that it overlaps with the periphery of the OEPD at around the time the inner ear begins to segregate. *Foxi1* remains absent from the putative otic placode, but is expressed in the EDP (Freter et al., 2008). One interpretation is that Foxi1 prevents commitment to the inner ear fate and that at early stages, both epibranchial and otic precursors do not express *Foxi1* and have the potential to form either cell type.

### INDUCTION OF THE INNER EAR

As stated above the development of the inner ear is progressive, involving the gradual restriction of cell fate, from non-neural ectoderm, to PPR, to OEPD and then to inner ear fate. This has been clearly demonstrated in the chick, using cultures of the putative inner ear ectoderm isolated and cultured *ex vivo*: ectoderm taken at mid-neurula stages (when the embryo has between 1 and 3 somites) can only express *Pax2*, an OEPD marker. In contrast ectoderm taken at late neurula stages (when the embryo has between 4 and 6 somites) can express *Soho1*, a transcription factor that is associated with commitment to the otic fate. Furthermore, ectoderm taken at this stage can form hair cells (Freter et al., 2008). This suggests that signals from surrounding tissue are still required to enable the transition from the OEPD to committed inner ear precursors.

*Wnt8a* (which is known as *Wnt8c* in the chick) is expressed in the caudal hindbrain is necessary for the partitioning of the otic placode from the OEPD (Freter et al., 2008). The application of FGF19 (the mesodermal signal in chick) and WNT8a to competent ectoderm was sufficient to complete inner ear development in non-neural ectoderm (Ladher et al., 2000). Inhibition of the Wnt signal using the antagonist Dkk1 blocked the expression of the inner ear marker *Soho1* but did not inhibit the expression of *Pax2* (Freter et al., 2008). This experiment provides further support of the progressive nature of inner ear induction. In mice, an inner ear specific mutation of β-catenin, a down-stream transducer of the wnt pathway, results a block in the formation of the inner ear. *Wnt8a* is induced in both chick and mouse as a result of FGF signaling. In chick, FGF19 can induce *Wnt8a* and in mice mutant for *Fgf3* fail to express *Wnt8a* (Urness et al., 2010). While these data suggest that *Wnt8a* in response to Fgf signaling is responsible for partitioning the inner ear from the OEPD, mouse mutants data presents a more confusing scenario. Mutant for *Wnt8a* do affect the otic placode (Vendrell et al., 2013). It is possible that other Wnts proteins expressed in the hindbrain, such as *Wnt1*, *Wnt3a,* and/or *Wnt6* are all able to compensate for the loss of *Wnt8*. Thus although Wnt signaling, as determined by the over-expression of the Wnt antagonist Dkk1 and the loss of the Wnt signaling transducer β-catenin, does result in a failure of the inner ear to partition from the OEPD, the significance of the most likely ligand, *Wnt8a*, is less clear. It is likely that it acts to ensure the inner ear forms in the correct axial position, however, other Wnt genes can compensate for its absence.

## MORPHOGENESIS

**FIGURE 2:**
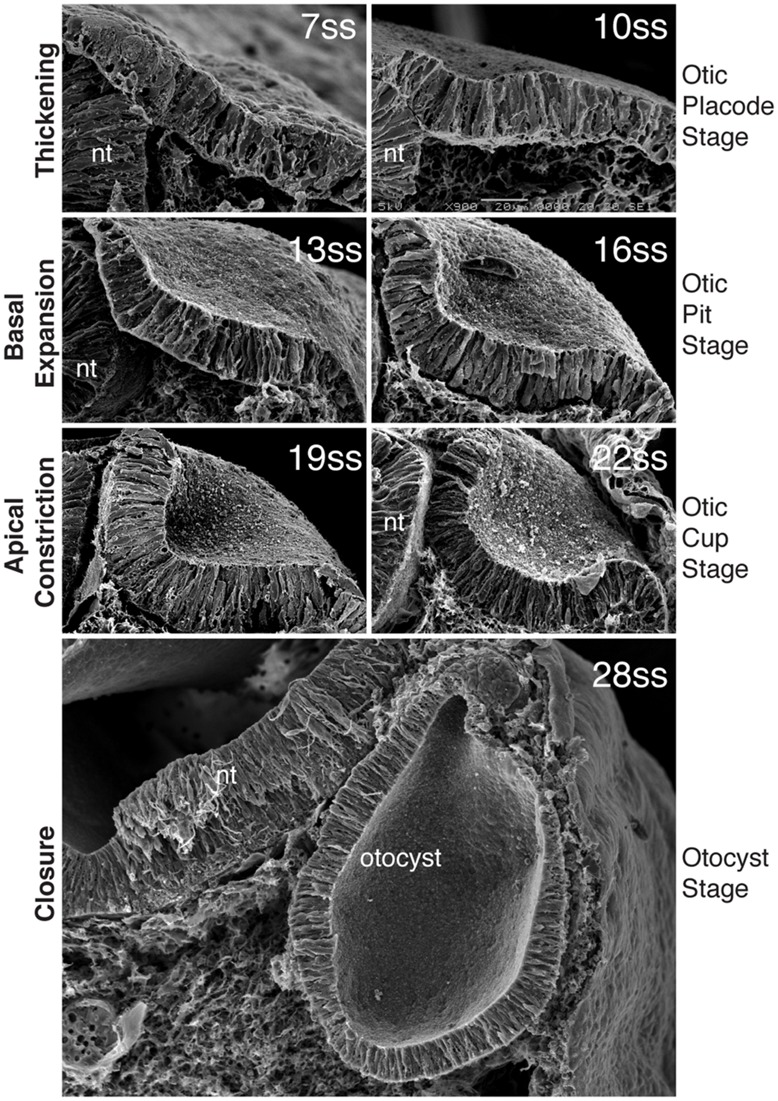
**Morphogenesis of the inner ear.** Scanning electron micrographs through the chick otic placode at different stages of development, show the stages of morphogenesis. By 7ss, the otic placode has segregated from the OEPD. At around 10ss of development, the placode begins to invaginates such that at 13ss and 16ss the placode forms a configuration known as the otic pit. Basal expansion predominates at this stage. After 16ss and until closure, apical constriction is the main processs driving invagination and the otic placode at these stages progressively deepens form the otic cup. With closure, the final form of the otocyst is apparent. (Modified from Sai and Ladher, 2008).

The inner ear is a closed structure located inside the head, embedded within the mesenchyme of the head. However, as described above, the placode is a superficial structure. From its position on the surface of the embryo, the otic placode must undergo a series of morphogenetic changes so that it ends up as an isolated closed vesicle in the cephalic mesoderm (Meier, 1978a,b). In this section, we describe the morphogenetic changes that take place as the otic placode changes shape to become the inner ear precursor (**Figure 2**). As the later morphogenetic events that shape the otocyst to the final inner ear are not well known, these will not be described.

### EPITHELIAL THICKENING

The otic placode itself already has a morphology that sets it apart from the rest of the ectoderm; it consists of a thickened columnar pseudo-stratified epithelium as opposed to the squamous-like epithelium surrounding it. In amphibians, chick and mouse, where this has been investigated, this thickening occurs around the time of otic induction, when the embryo has 4–6 somites. The mechanisms controlling the thickening of the placodal epithelium are not well known, although Pax2 has been proposed as a regulator of placodal thickening (Christophorou et al., 2010). The actual mechanics of placodal thickening are also unclear, however, studies investigating the function of members of the small GTPase family members, RhoA and Rac1 in the development of the lens placode suggest that Rac1 is a major effector of thickening (Chauhan et al., 2011) and is likely to be necessary for placodal thickening in the otic placode. Placodal thickening is likely to be necessary for the mode of proliferation in the inner ear. Cells within the otic placode divide by interkinetic nuclear migration (INM); Sauer, 1936). INM is thought to permit tighter packing of epithelial progenitors, which allows a higher concentration of cells (Fish et al., 2008). Furthermore, the packing density of pseudostratified epithelia is necessary for division. During INM, nuclei actively migrate apically to divide. Daughter nuclei are then passively pushed back to the basal side by the action of surrounding nuclei and by virtue of their packing density, and it is likely that if this packing density were not maintained division would be aberrant (Kosodo et al., 2011). It is also likely that thickened epithelia allow precise and separable control of both apical and basal domains of the placodal cells. As we discuss, this is particularly important in the control of invagination, in which signals act on the basal side and eventually result in apical changes.

### INVAGINATION

Soon after the partition of the otic placode from the OEPD, invagination begins to take place. By the time the embryo has between 8 and 10 somites, a slight depression in the epithelial plain is apparent. This depression continues to deform, deepening before pinching off and forming an enclosed vesicle within the cephalic mesoderm (**Figure 2**). The mechanisms behind otic invagination are only now starting to become clear. However, insights have been obtained using quite a simple analysis. Here, measurements of the apical and basal faces of the otic placode during its morphogenesis suggest two types of cell shape change (Alvarez and Navascués, 1990; Sai et al., 2014). Between 10 and 16 somites (in the chick), the apical face of the otic placode does not change, however, the basal side expands, the placode at this stage is also known as the otic pit. From 16 to 22 somites stages the apical length decreased dramatically, and the otic placode is driven deeper into the mesenchyme. The placode at this point adopts a configuration that has been termed the otic cup. Otic invagination itself can be considered as a biphasic process; phase one being basal expansion and phase two being apical constriction.

One of the driving forces behind changes in cell shape are alterations in the cytoskeleton, that is the network of actin fibers, microtubules, and intermediate filaments that provide structure and support to a cell (Lecuit and Lenne, 2007). Therefore, it is no surprise that during basal expansion clear difference in the distribution of actin are observed. At 10 somite stages (ss), actin fibers (or F-actin) can be detected in apically and basally in the otic placode cell. At 13ss, F-actin is depleted basally and enriched apically (Sai and Ladher, 2008). This is also when basal expansion occurs. Thus, the key to understanding basal expansion is to understand how basal F-actin is cleared. Furthermore, as basal expansion needs to occur in the whole of the otic placode, the changes in cell behavior must be coordinated across the tissue.

Clues about the mechanism came from experiments in which the otic placode was isolated and placed into culture. Explants of the otic placode, freed of underlying mesoderm and endoderm as well as adjacent neural tissue, are able to clear actin from the basal side and to round up and form otic vesicles if taken at 16 somites stage of development. However, otic explants taken at 10 somite stages of development neither clear actin from the basal side of the otic placode nor show any signs of epithelial deformation (Sai and Ladher, 2008). This suggests that extrinsic signals are required for invagination. One obvious candidate for the extrinsic signal that controls and coordinates invagination was the same signal controlling its induction, FGF. Indeed when 10ss explants were cultured with beads that locally delivered FGF the ability to clear basal actin was restored. Further experiments involving pharmacological inhibitors confirmed that the FGF pathway, through the activation of phospholipase C (PLC) gamma activated the motor protein myosin-II, by phosphorylating its regulatory subunit, myosin light chain. Active myosin-II exerted a non-canonical activity on the basal side that resulted in the depolymerization of F-actin. Thus, during basal expansion, active myosin light chain and F-actin are reciprocally localized; F-actin apically and phosphorylated myosin light chain basally. As we discuss later this is in contrast to apical constriction where both F-actin and active myosin-II co-localize to the apical face of the otic placode (Sai and Ladher, 2008).

While this mechanism for basal expansion has only been described during inner ear invagination, it is likely that it does occur in other epithelial tissues undergoing deformation. Prior to the elevation of the neural plate, the reciprocal localisation of phosphorylated myosin light chain and F-actin is also observed, suggestive of basal expansion (Sai and Ladher, 2008). Similarly, a transient reciprocal localisation is observed in the mesodermal progenitors about to invagination during *Drosophila* gastrulation (Dawes-Hoang et al., 2005; Fox and Peifer, 2007). In all cases it is likely that a signal acting on the basal side of the cell triggers this activity, ensuring not only a coordination amongst all of the cells within the tissue, but also direction to the deformation. With respect to *in vitro* tissue engineering approaches, this does suggest that signals need to be spatially confined and care must be given to which side of the cell is stimulated by ectopic signals.

From 16 somites stage of chick development, the apices of the otic placode cell begin to constriction, deepening the invagination and driving the internalization of the otocyst. The mechanisms of apical constriction are similar to those that have been determined for other epithelial remodeling events such as wound healing, neural tube formation, or dorsal closure in *Drosophila* (Sawyer et al., 2010; Martin and Goldstein, 2014). During otic morphogenesis, this phase is characterized by the co-expression of active myosin II and F-actin (Sai et al., 2014). Thus the key to understanding apical constriction in the otic placode is to understand how myosin II is activated apically.

Recent findings suggest that the mechanisms of apical constriction during otic invagination are analogous to those driving neural tube closure (Nishimura et al., 2012). Apical constriction is powered by the canonical activity of the myosin-II motor generating contractile tension in actin filaments. This activity is localized circumferentially in the apex of the cell, and is coincident with the apical junctional complex (AJC). The AJC includes both tight junctions and adherens junctions, which act to maintain the integrity of the epithelia. These junctions act as anchor points from actomyosin fibers, and it is from here that force can be more effectively transmitted across cells. Thus the activation of the myosin-II motor occurs here (Nishimura et al., 2012; Sai et al., 2014). The RhoGTPase family has been frequently implicated in the regulation of junctional actin by activating Myosin-II, and has been shown to be important in the regulation of apical constriction in many other systems (Martin and Goldstein, 2014). RhoGTPases act as switches, cycling between a GTP-bound “ON” state and a GDP-bound “OFF” state. This switch is control by the action of effectors of RhoGTPases; Rho Guanine exchange factor (RhoGEF) switches on Rho, and a Rho Guanine Activating Protein (RhoGAP) switches it off (Hall, 2012) and thus it is likely that upstream control of Rho activity is exerted by specific GEFs and GAPs. For example, studies in *Drosophila* gastrulation have shown that RhoGEF1 stimulates Rho1, which in turn activates the *Drosophila* homolog of the Rho-associated, coiled-coil protein kinase (ROCK). Drok is responsible for the activation of myosin-II (Mason et al., 2013). Similarly, in vertebrate neurulation RhoA activation by the GEF ArhGEF11 is necessary for neural tube closure through the activation of myosin light chain (Nishimura et al., 2012). In the otic placode, RhoA is localized apically and is similar to neural tube closure, is also activated by ArhGEF11 (Sai et al., 2014). Once active, RhoA is able to activate ROCK, which directly phosphorylates myosin light chain, thus activating myosin-II. This network, is likely to be conserved across a range of epithelial morphogenetic systems, and ensures apically restricted myosin-II.

One further consideration is the separation of basal expansion and apical constriction. ArhGEF11 is expressed at around 13ss, coincident with apical constriction. Furthermore it is localized to the otic placode, and is not found in surrounding epithelia, suggesting that its expression is likely to be regulated by otic induction cues (Sai et al., 2014). Thus, it is probable that signaling is not only responsible for phase 1 otic morphogenesis but also, indirectly, for phase 2. In phase 1 morphogenesis, actin is cleared from the basal side of the otic placode, through the direct action of FGF signaling, ensuring that the contractive activity of myosin-II and RhoA activity cannot act ectopically (Sai and Ladher, 2008). In phase 2, the induction and expression of ArhGEF11 activates RhoA and the thus myosin-II. The combination of RhoA and Myosin-II activity permits the contractive activity of this motor protein, and mediates apical constriction.

Once internalized the otic vesicle must then be closed off, with the two edges fusing, isolating the inner ear vesicle from the rest of the ectoderm. The mechanism of fusion is still not clear, but parallels can be drawn with wound healing and with the closure event that occurs once the neural tube has formed a tube (Colas and Schoenwolf, 2001; Martin and Parkhurst, 2004). Preliminary data does suggest that the edges of the otic epithelium send out protrusive processes, and these filopodia knit the knit the epithelium together such that it is now contiguous. Once closed, the isolated otocyst now is primed to undergo further shape changes to form the final structure of the membranous labyrinth. These morphogenetic mechanisms are only now beginning to be understood and are described elsewhere (Chang et al., 2004; Mansour and Schoenwolf, 2005; Abraira et al., 2008; Ohta et al., 2010). These processes may employ similar mechanisms or utilize extrinsic signals, also acting in a localized and directional fashion, to complete the final maturation of the inner ear.

## CONCLUSION

Both induction and the early morphogenesis of the inner ear require signaling factors deployed in both a spatially and temporally restricted pattern. Mimicking these signals *in vitro* to generate *in vitro* inner ears may at first pass seem daunting. However, the therapeutic value in these studies will come from understanding the spatial and temporal effect of signaling and then to employ them when needed. It should be noted that for the differentiation of hair cells and of otic placode derived neurons, only the initial signals need to be recapitulated (Freter et al., 2008). Once the otic placode has been induced hair cell and neurons will differentiate without the need for further tissue interactions and additional signals. These kinds of ideas have formed the basis of differentiation protocols that steer ES cell fate to otic placodal fates, however, their efficacy as potential therapies remains to be validated rigorously (Li et al., 2003; Oshima et al., 2010; Chen et al., 2012; Koehler et al., 2013). In some of these differentiation protocols, *in vitro* generated otic progenitors have been used to repair lesions in the auditory nerve (Chen et al., 2012). These studies although promising, highlight the necessity to further improve and build on our knowledge of inner ear induction and early morphogenesis so that these finding can be used to deliver more efficient and effective therapeutic *in vitro* generated inner ear tissues, that many next-generation therapies will utilize.

## Conflict of Interest Statement

The authors declare that the research was conducted in the absence of any commercial or financial relationships that could be construed as a potential conflict of interest.
